# Abstracts

**Published:** 1913-09

**Authors:** 


Philip F. Williams and Richard M. Pearce, *Surg. Gyn. and Obs.*, April, 1913, have employed the Aberhalden method in 28 cases of pregnancy and 8 post partum cases, including 1 after abortion. The test has never been negative in a known pregnancy, but the serum of pregnancy reacts with kidney, heart and uterus and even with non-human tissues (*e. g.*, dog's kidney). Like Schwartz, they believe the nonhydrin test superior to the biuret reaction. They consider that results as satisfactory as with dialysis, may be obtained by mixing tissue and serum in tubes, incubating for 24 hours, coagulating with heat and acetic acid and applying the ninhydrin test to the filtrate. But they hold that the test should not be accepted clinically until it has been further investigated. Carey Pratt McCord of Detroit, ibid., basing his conclusions on 240 tests under various conditions and at different periods of pregnancy, considers the test as reliable and practical but subject to limitations and with the proviso that the technic is accurately followed.

